# Treatment of Acute Promyelocytic Leukemia with High White Cell Blood Counts.

**DOI:** 10.4084/MJHID.2011.038

**Published:** 2011-09-08

**Authors:** C. Kelaidi, L. Adès, P. Fenaux

**Affiliations:** 1 Department of Hematology, G. Papanikolaou Hospital of Thessaloniki, Exochi 57010, Greece; 2 Service d’Hématologie, Hôpital Avicenne - Université Paris 13, 125, rue de Stalingrad 93000 Bobigny, France

## Abstract

Acute promyelocytic leukemia (APL) with WBC above 10 G/L has long been considered, even in the all-trans retinoic acid (ATRA) era, to carry a relatively poor prognosis (compared to APL with WBC below 10 G/L), due to increased early mortality and relapse. However, early deaths can to a large extent be avoided if specific measures are rapidly instigated, including prompt referral to a specialized center, immediate onset of ATRA and chemotherapy, treatment of coagulopathy with adequate platelet transfusional support, and prevention and management of differentiation syndrome. Strategies to reduce relapse rate include chemotherapy reinforcement with cytarabine and/or arsenic trioxide during consolidation, prolonged maintenance treatment, especially with ATRA and low dose chemotherapy, and possibly, although this is debated, intrathecal prophylaxis to prevent central nervous system relapse. By applying those measures, outcomes of patients with high risk APL have considerably improved, and have become in many studies almost similar to those of standard risk APL patients.

## Introduction:

Acute promyelocytic leukemia (APL) is a distinct type of acute myeloid leukemia (AML) characterized by specific morphology (M3 in the FAB classification), frequent association of a coagulopathy, the t(15:17) translocation resulting in the fusion protein PML-RARα. The APL has a specific sensitivity to the differentiating properties of all-trans retinoic acid (ATRA) and the proapoptotic effect of arsenic trioxide (ATO), which have, in combination with anthracyline-based chemotherapy, considerably improved its prognosis in the last 20 years.^1^–^6^

WBC counts are generally low in APL, with only 20–25% and <5% of patients having WBC higher than 10 G/L and 50 G/L, respectively.^7^–^11^ High WBC counts have always been associated with poorer prognosis in APL, even since the advent of ATRA, but recent results suggest that the outcome of those patients may have become almost similar to that of APL with lower WBC counts, with the optimal use of all therapeutic tools, including early massive transfusional support.

## Characteristics and prognosis of APL with increased WBC counts:

22.6%, 6.1% and 1.4% of 902 patients, included in combined analysis of APL 93 and APL 2000 trials of the European APL group, had WBC 10–50 G/L, 50–100 G/L and >100 G/L, respectively.^12^ The 10 G/L and 50 G/L thresholds for WBC are used hereafter to define APL with high and very high WBC. APL with high WBC is more frequent in children and is often associated with the microgranular variant (M3v), with the short PML-RARα isoform (bcr3), with the FLT3 mutation and the CD56 expression which are all adverse prognostic factors in APL, although not independent from WBC counts.^13^–^18^ APL with increased WBC is frequently associated with severe coagulopathy and, sometimes with organomegaly. The fibrinogen level is indeed <1.5 g/L in at least half the patients with increased WBC and is often accompanied by severe hemorrhagic manifestations, intracranial hemorrhage either at presentation or during the first days of remission induction obviously being the most severe. Before the ATRA era, i.e. in patients treated with anthracycline-based chemotherapy, early mortality in patients with increased WBC averaged 50–70%, but even in the early ATRA era, most studies found an increased early mortality (10–20%) in patients with high WBC counts.^7^,^19^–^21^ They also found, by comparison with patients with WBC<10 G/L, an increased incidence of relapse (30%), including of central nervous system (CNS) relapse (Tables 1 **and** 2).^8^,^9^,^22^–^28^ This increased relapse risk led to the design of Sanz score for APL relapse, discriminating between low and intermediate risk (hereafter referred to as standard risk) patients with WBC <10 G/L and platelet counts more and less than 40 G/L, respectively, and high risk patients with WBC counts greater than 10 G/L.^29^ In that study, with an ATRA and anthracycline-based regimen, 3-year relapse-free survival was 100%, 90% and 75% for low, intermediate and high risk patients, respectively. Thus, preventing relapse is particularly relevant for high risk patients. In addition, considering that event-free survival (EFS) and OS are probably better after salvage treatment delivered at the molecular relapse stage (rather than at full blown hematologic relapse), monitoring of PML-RARα after CR achievement is particularly important for high risk patients.^30^–^32^

## Management of APL with Increased WBC Counts

-1) *Preventing early mortality:* Hyperleukocytic APL is, even more than standard risk APL, a medical emergency. An undetermined percentage of patients with high and very high WBC die before treatment onset without being registered in clinical trials.^33^ Early mortality rates as high as 42% have been reported in APL patients with high WBC, many of those deaths occurring before patients could reach a specialized hospital facility, let alone be included in a clinical trial, while in a report from Surveillance, Epidemiology, and End Results (SEER) an overall early mortality rate of 17% was reported in APL, which was presumably more in patients with high WBC counts.^34^,^35^ The risk of early mortality being particularly high during the first days of treatment, specific measures must be urgently implemented to prevent those early fatalities. Resistance to ATRA-based induction regimens being very rare (<1/500 cases), induction remission failures actually reflect early mortality. Published trials of the modern era reporting on early mortality of high risk patients are listed in Table 1 although, as said above, they may sometimes underestimate the reality, some patients possibly dying before they can enter a clinical trial.

Bleeding, particularly in the CNS, is the leading cause of early and very early (<48 h) mortality. Presence of coagulopathy, high WBC and higher than normal creatinine levels have been identified as predictive factors of fatal hemorrhage.^36^,^37^ Coagulopathy should be treated intensively with fresh frozen plasma and high dose prophylactic platelet transfusions in order to maintain platelet counts ≥50 G/L until normalization of bleeding parameters. In accordance with published guidelines, invasive procedures including placement of central venous catheters and leukapheresis are contra-indicated during induction remission.^34^ Any delay in diagnosis and treatment initiation in hyperleukocytic APL is unequivocally associated with a higher risk of early hemorrhagic death.^38^ ATRA has a rapid impact on both fibrinolytic and procoagulant aspects of the coagulopathy, and should be initiated as soon as the diagnosis of APL is clinically or morphologically suspected. However, at least in hyperleukocytic forms, chemotherapy should be started concomitantly, in order to avoid a life threatening differentiation syndrome (see below). This concomitant use of ATRA and chemotherapy had led to reduction of early mortality in patients with very high WBC (>50 G/L), from at least 50% before the ATRA era to 18% in the APL93 trial.^8^

Differentiation syndrome (DS) with ATRA, with manifestations of unexplained respiratory failure, pulmonary infiltrates, fever, weight gain, pleuropericardial effusions, hypotension and renal failure, usually occurring concomitantly with a rise in WBC count, is another major cause of early mortality in high risk patients.^39^,^40^,^41^ Severe DS, requiring mechanical ventilation or complicated with pericarditis or life threatening hemorrhage, is more frequent in patients with high presenting WBC counts. Early recognition and systematic prevention of DS with high-dose dexamethasone from the first day of treatment certainly contributed to reduce the number of fatal DS from 5.7% to 3.9%, among high risk patients included in two consecutive trials of the European APL group (APL 93 and APL 2000).^12^

Overall, improved DS management and transfusional support were likely the key factors of reduction of early mortality between our APL 93 and 2000 trials, from 10.4% to 7% in patients with high WBC counts, and 18% to 9% in patients with very high WBC counts. In APL 2000 trial, the early death rate was not dependent from WBC count.^12^ Moreover, in our ongoing APL 2006 trial, using the same induction regimen of ATRA and anthracycline based chemotherapy (with substitution of idarubicin for daunorubicin), none of the 45 high risk patients included had early death.^42^

-2) *Preventing relapse:* Presenting WBC >10 G/L remains the strongest prognostic factor for relapse, including for extramedullary relapse, especially in CNS. Several strategies can reduce the incidence of relapse in those patients, and attenuate the adverse prognostic character of hyperleukocytic APL, which was no more significant in our recent experience.

a) Cytarabine during induction and consolidation treatment: While anthracycline-based regimens without AraC have been preferred to limit toxicity in low and intermediate risk APL patients, several lines of evidence strongly support that cytarabine added to anthracycline during remission induction and consolidation treatment may contribute to reduce relapses in high risk patients.

Indeed, very few relapses occurred in our APL 2000 trial and in a German study using high dose cytarabine, added to anthracyclines, during consolidation, (Table 2).^9^,^24^,^43^ In addition, a joint analysis of the European group trial APL 2000 and the Spanish PETHEMA trial LPA 99, which used no cytarabine during induction and consolidation, showed a significant advantage, in terms of EFS, CIR and OS in high risk patients included in APL 2000 trial.^44^ Finally, risk-stratification in the subsequent PETHEMA-HOVON LPA 2005 trial, based on reinforcement of consolidation with cytarabine in high risk patients, reduced relapse rate by comparison with LPA99 trial.^26^

The dose of cytarabine could also be of importance. Increasing the total cytarabine dose from 8 to 20 g/m2 during the second consolidation cycle indeed possibly contributed to the lower incidence of relapse of patients with high and very high WBC count included in APL 2000, compared with APL 93 trial.^12^ Moreover, long term analysis of our APL 2000 trial found that outcome of high risk patients was better than that of standard risk patients treated without cytarabine.^43^

b) Maintenance treatment: In our experience, maintenance treatment with continuous low dose 6MP+MTX and intermittent ATRA is particularly useful in high risk patients. Its impact on the long-term outcome was clearly demonstrated in the very long term analysis of the randomized APL 93 trial where 10-y CIR was 68.4%, 53.1%, 32.8% and 20.6% in patients with WBC >5 G/L who had received no maintenance, maintenance with only ATRA, only chemotherapy (6MP and MTX) and combined maintenance with ATRA+chemotherapy, respectively (P<0.001). The difference was less important for patients with WBC <5 G/L, suggesting that combined maintenance treatment mostly benefited high risk patients.^22^ Moreover, a combined analysis of APL 93 trial (where not all patients received maintenance) and APL 2000 trial, where combined maintenance treatment was systematic, showed that, in patients with WBC count >10 G/L, receiving combined maintenance treatment was the strongest prognostic factor associated with reduction of CIR and increase in OS.^12^ We also found discontinuation of this maintenance treatment after less than one year to be associated with an increased risk of relapse. Two-year combined maintenance with intermittent ATRA and low dose chemotherapy thus appears to further improve the outcome of hyperleukocytic APL. This treatment is associated with no excessive toxicity, provided co mplete blood count is regularly monitored to adjust doses and avoid excessive cytopenias.

c) Arsenic trioxide: Arsenic trioxide is the most potent single agent in APL, capable of inducing complete responses, including molecular ones, and it is particularly successful in the setting of molecular and hematologic relapse.^32^ A US intergroup trial clearly demonstrated the benefit of adding two cycles of single agent ATO to a classical first line ATRA and chemotherapy based regimen, particularly in high risk patients.^24^ In addition, 5-year EFS of high risk patients who received ATO was not significantly different from that of standard risk patients, indicating that ATO consolidation may overcome the negative prognosis conferred by high risk disease. Other studies using ATO during consolidation also generated promising results (Table 2).^45^–^47^ In contrast, induction with ATO monotherapy is considered more experimental, in particular due to the fact that high risk patients have a major risk of developing severe DS, unless chemotherapy is administered concomitantly.^48^,^49^ Nevertheless, once CR is achieved, durable responses have been reported with this approach.

Thus, although ATO may become frequently used in the front-line induction therapy of APL, published results caution against using it without chemotherapy in high risk patients. In its current randomized APL 2006 trial, the European APL group is investigating the impact of ATO versus that of AraC during consolidation in high risk patients.^42^

d) Prevention of CNS relapse: High WBC count is also a major risk factor for extramedullary relapse, in particular CNS relapse, although its incidence is small (cumulative incidence of 5% among high risk patients).^51^,^52^ Given the published poor prognosis of extramedullary relapse,^51^ CNS prophylaxis with intrathecal chemotherapy may be considered systematically in high risk patients. Agents that cross the blood-brain barrier such as high dose cytarabine or arsenic trioxide (ATO) may also reduce that risk. In APL 93 trial, using high dose cytarabine, 3 (0.9%) patients in CR had CNS relapse as compared with 15 (2.5%) and 9 (2.1%) patients in AIDA 0493 and AIDA 2000 trials, respectively, where the dose of AraC was conventional.^22^,^27^,^28^ Moreover, systematic CNS prophylaxis with 5 triple intrathecal injections, in addition to higher dose cytarabine, in patients with high WBC count in APL 2000 trial was associated with the absence of CNS or other extramedullary relapse. The German AMLCG study using higher dose cytarabine also reported no CNS or other extramedullary relapse in standard and high risk patients.^24^ Despite penetration in cerebrospinal fluid, prolonged use of ATO did not seem efficacious in preventing CNS relapse in the absence of intrathecal chemotherapy in one study, where all 4 relapses (among 80 patients) involved the CNS.^50^

However, other authors consider the number of high risk patients to treat prophylactically too high given the overall low incidence of CNS relapse.^52^ Also of note is that, since the advent of ATO to treat relapse, outcome of patients with CNS relapse has been better in our experience than with previously used salvage regimens.^53^

-3) *APL with high WBC counts in specific age groups:*

a) Children: The frequency of high WBC counts in children is relatively high and even more (50%) in children aged <12 years.^13^–^16^ In our experience, children aged <4 years have a higher risk of relapse even with prolonged maintenance treatment and high dose AraC.^16^ For older children, EFS did not differ from that of adults after adjustment for WBC counts, and OS was often better due to better results of salvage treatment.^14^ High risk pediatric patients included in Italian trials had a 10-year EFS of 59%, significantly worse than in standard risk patients, while the Spanish group reported an overall 5-year CIR of 31% in children with high WBC counts. By contrast, similar outcomes were reported in high and standard risk patients in a Japanese study using cytarabine, added to ATRA and anthracyclines.^54^

ATO, in combination to ATRA and chemotherapy, may offer an interesting perspective for disease control in pediatric patients with high WBC counts, especially in those aged <4 years, associated in our experience to a higher relapse risk. No chronic arsenic toxicity was observed in a Chinese report on childhood APL, despite protracted intermittent administration of ATO used as a single agent.^55^

b) Elderly patients: Approximately 20% of APL patients are aged over 60 years, with a proportion of cases with WBC >10 G/L slightly lower than in younger adults (20% vs. 23% high risk patients among those aged ≥60 years vs. 19–59 years, respectively, in the PETHEMA studies).^56^–^58^ Increased incidence of early deaths and deaths in CR in elderly APL patients in general renders particularly challenging the management of those patients, especially those with high risk features. Indeed, in both the GIMEMA and PETHEMA studies, one third of elderly patients died during induction remission.^56^,^57^ Early deaths were due to hemorrhage and DS but also to cardiac complications.^56^ Relapse rate of high risk elderly patients is similar to that of high risk younger patients.^58^ Age above 50 years, in spite of absence of CIR difference across age groups, was, however, an adverse prognostic factor for EFS and OS in high and very high risk patients, in the combined analysis of APL 93 and APL 2000 trials.^12^ Rather than age-adjusted chemotherapy dosage, incorporation of ATO in current regimens could counteract those adverse features with better disease control.

## Conclusion:

Improvement in outcomes of APL patients with high WBC counts was made possible by combining specific measures to prevent early mortality and relapse. Those results indicate that, with risk-tailored management, outcome of APL patients could become independent of WBC counts in the Acute promyelocytic leukemia. More investigation and efforts are still needed to reduce very early mortality not reflected by clinical trials results.

## Figures and Tables

**Table 1.** Early mortality of high risk (HR) patients in modern era clinical trials according to the type of induction treatment.

***Study***	***Years of study***	***Nb of HR pts***	***Early death rate (%)***
***ATRA/anthracycline, with standard dose AraC***			
Fenaux et al APL 93^8^	1993–1998	139	12.2%
Adès et al APL 2000^9^	2000–2006	133	7.4%
Adès et al APL 2006^42^	2006-…	45	0
Powell et al C9710^23^	1999–2005	113	20%
***ATRA/anthracycline with high-dose AraC***			
Lengfelder et al AMLCG^24^	1994–2005	37	16.2%
***ATRA/anthracycline without AraC***			
Sanz et al LPA99^26^	1996–2005	140	19%
Sanz et al LPA2005^27^	2005–2009	118	17%
***ATO monotherapy***			
Ghavamzadeh et al^49^	1999–2010	37	42%
***ATO+/−ATRA+chemotherapy***			
Hu et al (ATRA/ATO/chemotherapy)^50^	2001–2005	19	5%
Ravandi et al (ATRA/ATO/gemtuzumab ozogamycin or idarubucin)^47^	2002–2008	47	19%

**Table 2.** Published clinical trials reporting long-term outcome in high risk (HR) patients according to the type of consolidation.

***Study***	***Years of study***	***Nb of HR pts***	***Relapse***	***OS***
***AraC-containing consolidation***				
Adès et al APL 9322^9^	1993–1998	139	WBC>5 G/L: 10y-CIR 37.8%	*WBC>5 G/L: 10y-OS 63*.1%
Adès et al APL 2000^9^	2000–2006	133	5y-CIR 7.5%	5y-OS 89.8%
Lengfelder et al AMLCG^24^	1994–2005	37	10y-CIR 11.4%	10y-OS 73%
Grimwade et al AML15^31^	2002–2009	55	3y-CIR 10% (+/− AraC)	-
***Risk-stratification consolidation***				
***AraC added to ATRA/anthracycline for HR patients***				
Sanz et al LPA99^26^	1996–2005	140	4y-CIR 27%	4y-OS 68%
Sanz et al LPA2005^27^	2005–2009	118	4y-CIR 14%	4y-OS 79%
***ATRA added to AraC/anthracycline for HR patients***				
Lo-Cocco et al AIDA-0493^28^	1993–2000	176	6y-CIR 49.7%	6y-OS 61.3%
Lo-Cocco et al AIDA-2000^29^	2000–2006	129	6y-CIR 9.3%	6y-OS 83.4%
***ATO studies***				
***ATO monotherapy***				
Mathews et al^48^	1998–2004	17	5y-EFS 60%	-
Ghavamzadeh et al^49^	1999–2010	38	All risks: 5y-DFS 66.7% No difference between HR pts and others	All risks: 5y-OS 64.4% No difference between HR pts and others
***ATO+chemotherapy***				
Hu et al (ATRA/ATO/chemotherapy)^50^	2001–2005	19	5y-EFS 83.2%	5y-OS 89.2%
Ravandi et al (ATRA/ATO/gemtuzumab ozogamycin or Idarubicin)^47^	2002–2008	47	3y-EFS 60%	3y-OS 60%
Powell et al ATO+^23^	1999–2005	55	5y-EFS 60%	5y-OS 80%
Powell et al ATO−23	1999–2005	58	5y-EFS 35%	5y-OS 40%
